# Predation and reproductive performance in two pelagic typhloplanid turbellarians

**DOI:** 10.1371/journal.pone.0193472

**Published:** 2018-03-14

**Authors:** Arnola C. Rietzler, Henri J. Dumont, Odete Rocha, Marcela M. Ribeiro

**Affiliations:** 1 Department of General Biology, Federal University of Minas Gerais, Minas Gerais, Brazil; 2 Institute of Hydrobiology, Jinan University, Guangzhou, China; 3 Department of Ecology and Evolutionary Biology, Federal University of São Carlos, São Paulo, Brazil; University of Shiga Prefecture, JAPAN

## Abstract

We investigated feeding and reproductive performance of coexisting pelagic turbellarians from experiments on predation rates of *Mesostoma ehrenbergii* and *M*. *craci* as a function of food (*Daphnia similis*, three levels) and temperature (4 levels) during 10 days. Flatworms were collected from the pelagic of a subtropical lake in Brazil. Growth was more rapid at higher temperatures: more prey were consumed, and more eggs produced. *M*. *craci* and particularly *M*. *ehrenbergii* fitted a linear mixed-effects model and showed a type II functional response. *M*. *craci* was the more stenothermic of the two. Intrageneric predation also occurred: *M*. *ehrenbergii* fed on *M*. *craci*, but not vice versa. After a first clutch of subitaneous eggs, *M*. *ehrenbergii* produced resting eggs only. In *M*. *craci* an intermediate type of eggs hatched some time after release, survived passage through the gut of *M*. *ehrenbergii*, but did not resist drying. By primarily selecting cladoceran prey, *M*. *ehrenbergii* can make coexistence of both flatworms possible. As population density of *M*. *ehrenbergii* increases, it turns to producing resting and non-viable subitaneous eggs, thus limiting its population size. In nature, these processes structure the zooplankton community, while avoiding extinction of prey and predator.

## Introduction

That predation structures freshwater plankton communities was documented in the nineteen sixties, based on the effect of fish on zooplankton [1]. Zooplankton size structure shifts towards small-sized species under vertebrate predation and to large-sized species under invertebrate predation [2].

Later, it was shown that predation not only changes community composition by local extinction of prey [3, 4], but it also modifies prey morphology, escape behavior, reproductive traits and population dynamics [5–9].

Studies on invertebrate predators have focused mainly on carnivorous cladocerans and copepods in temperate regions [10,11] and on cyclopoid copepods and chaoborids in the tropics [7,12,13]. However, tropical freshwaters frequently shelter other invertebrate carnivores, such as medusae (Cnidaria), water mites and typhloplanid turbellarians. These too may structure zooplankton communities [14–16].

Despite an interest in flatworm predation [17–19] their migratory behavior [20,21] and feeding strategies [22,23], aspects of their biology, such as predatory interactions among co-existing species [24] have not been well explored.

*Mesostoma* spp. of the family Typhloplanidae produce two types of eggs [25]. Subitaneous eggs are common at first reproduction, resting eggs appear later [26]. Here, we investigate predation rates (functional response) of *Mesostoma ehrenbergii* and *M*. *craci* at different prey densities (2, 5 and 10 individuals per 80 mL) and temperatures (20, 24, 28 and 32°C) during 10 days. Such prey types, densities and temperatures occur in natural conditions [27, 28], with daphnids the favorite cladoceran prey. Our experimental study quantified feeding and reproductive performance of two species that co-occur in the pelagic and feed upon each other as well.

## Material and methods

### Origin and maintenance of the turbellarians and cladocerans

*M*. *ehrenbergii* and *M*. *craci* were collected from Lake Jacaré (19S 48´38´´, 42W 38´55´´), Rio Doce Valley, Minas Gerais, Brazil, in 2012. The lake is situated on private land with research consent to a Long Term Studies Program (PELD/ CNPq, site 4). It is not located in a protected area and no permits are required. After being accidentally collected by a plankton net, the turbellarians were isolated and maintained in laboratory cultures, aiming at investigative studies on this neglected group. The two species differ in size: *M*. *ehrenbergii* reaches up to 0.6–0.7 cm at 25°C; *M*. *craci* is around one third that size. Cultures had neutral pH, conductivity of 160.0–180.0 μS cm^-1^, dissolved oxygen around 6.0–7.0 mg L^-1^ and hardness between 32.0–36.0 mgCaCO_3_ L^-1^. They were maintained at 12h photoperiod, 24±1°C, and fed Daphnia *ad libitum*.

Parthenogenetic females of *Daphnia similis* were obtained from stock cultures maintained for ecotoxicological studies, as described above, fed *Raphidocelis subcapitata* (10^5^ cells mL^-1^) and a compound diet of fish food plus yeast (1mL L^-1^) [29].

### Predation of *Mesostoma* spp. on *D*. *similis*

The flatworms were acclimated for a generation to all temperatures tested and fed *D*. *similis*. Prey were similarly acclimated. Afterwards, newborn predators were individually transferred to beakers containing 80 mL of water and fed 2, 5 and 10 prey at 20, 24, 28 and 32 ± 1°C. Per treatment, three replicates for *M*. *craci* and four for *M*. *ehrenbergii* were applied. In *M*. *craci* experiments, prey offered were neonates (less than 24h old, 0.50–0.70mm in size); in *M*. *ehrenbergii*, *D*. *similis* were 3 days old, measuring around 1.50–1.80mm. Prey consumption counting as well as prey and medium replacement were done daily during 10 days. At the end of the experiments, the total number of eggs produced (for all replicates) was registered. In addition, the relationship between prey consumed and offered, was used to verify the type of functional response in both predators [30].

### Intrageneric predation by *Mesostoma* spp.

We applied 6 treatments in test-recipients of 50 mL at 24±1°C, with replacement, during 5 days as shown in Table 1.

**Table 1:** Intrageneric predation by *Mesostoma* spp. at 24±1°C, 6 treatments across 5 days.

Treatments	*Mesostoma ehrenbergii*	*Mesostoma craci*
**1**	1 adult*	1 adult*
**2**	1 adult	3 adults
**3**	1 young**	1 young**
**4**	1 young	3 young
**5**	1 young	1 adult
**6**	3 young	1 adult

* adult: over ten days old.

** young: three-four days old.

Both flatworm species were separately kept in controls at the same densities. Eggs of adult *M*. *craci* were collected to evaluate hatching percentage and time to hatching after release in the controls (n = 16) and after passing the digestive tract of *M*. *ehrenbergii* in treatments 1 and 2 (n = 21).

An additional experiment evaluated resistance of eggs to desiccation. The eggs (n = 20) were collected from cultures kept at 24±1°C. Eggs were dried at room temperature (around 25°C) for around 5 days, rehydrated and possible hatching observed for several weeks.

### Statistical analysis

Predation rate was modeled by fitting linear mixed-effects models, considering time (days) as a random-effect, and prey density and temperature, plus their interaction, as fixed-effects. The pairwise difference in the estimated parameters for each combination of prey density and temperature was tested using a 5% Tukey-adjusted significance level. Residual plots were checked to confirm no deviation from homoscedasticity or normality. We used the ‘lmer’ function from the ‘lmer4’ package[31] and ‘lsmeans’ function from ‘lsmeans’ package [32], both from R statistical software [33].

Temperature and prey density effects on egg production by both predators were analyzed by two-way ANOVA. Total of eggs produced was log transformed in the case of *M*. *ehrenbergii*. When significant, a Tukey test was applied.

Differences in predation rates in the intra-*Mesostoma* experiment were analyzed by Kruskal Wallis test, followed by a Dunn test. Egg hatching rates of *M*. *craci* were analyzed by Kruskal Wallis test and time for hatching by T- test, where homocedasticity and normality were checked (PAST version 3.0 [34]).

## Results

**Fig 1:**
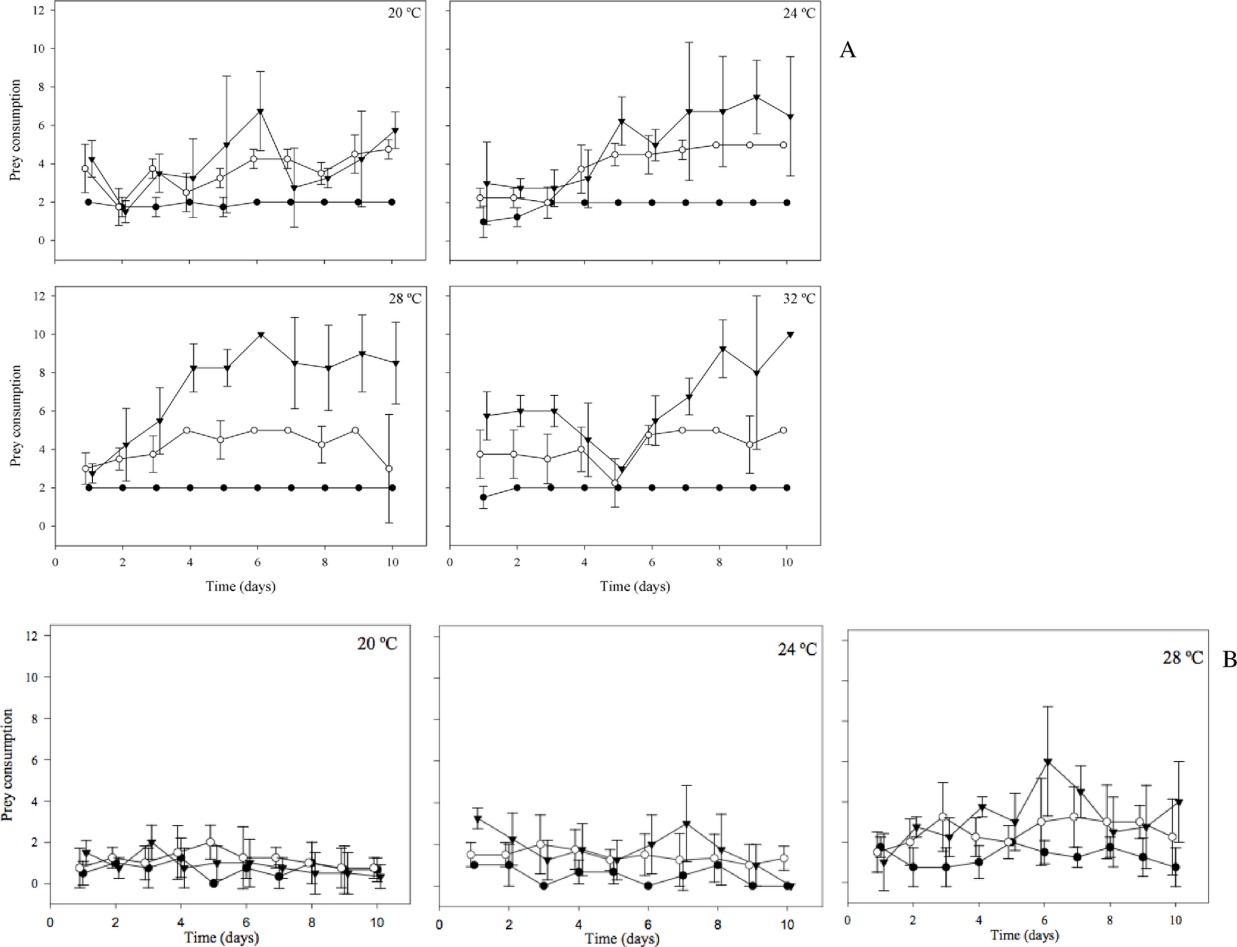
Daily mean consumption of *Daphnia similis* by *Mesostoma ehrenbergii* (A) and *Mesostoma craci* (B) at different temperatures and prey densities during 10 days. Closed circle, open circle and closed triangle correspond to 2, 5 and 10 prey, respectively.

Daily mean predation rates for both species are shown in Fig 1. For *M*. *ehrenbergii*, the lowest prey density resulted in total consumption. At the other densities (5 and 10), consumption increased with prey availability. At those prey densities and at 20 to 32°C there was a tendency for prey consumption to increase up to the 6th– 7th day, followed by a leveling-off towards the end of the experiment. Irregular fluctuation in prey consumption rates of *M*. *ehrenbergii* occurred in the combination of highest temperature and highest food level.

The best fit for mean predation rates across 10 days, a linear mixed-effect model, is shown in the supporting information (S1 Fig). The best fit for *M*. *ehrenbergii* was obtained at 24°C (*p* values significant at all prey densities) (S1 Table). In general, lower and higher temperatures did not cause daily differences in consumption at the prey densities considered. At lower densities of *M*. *craci*, there were no significant differences in daily mean predation rates at 20 and 24°C. A better fit was obtained at 24°C for 5 and 10 prey. No fit was found at 28°C.

*M*. *craci* responded less clearly to prey availability and temperature, and had lower prey consumption than *M*. *ehrenbergii* (Fig 1). Feeding rates were lower and more variable, especially at 20 and 24°C. At 28°C, *M*. *craci* showed a similar predation pattern as that of *M*. *ehrenbergii*, despite lower rates of prey consumption.

On the other hand, 2 prey were always totally consumed by *M*. *ehrenbergii*, while 10 prey provided excess food at lower temperatures (20 and 24 ± 1°C) and enough food for growth and/or egg production at 28 and 32 ± 1°C across 10 days. Mean prey consumption at higher prey densities (5 and 10) varied from 3.62 to 4.02 at 20°C; 3.90 to 5.05 at 24°C; from 4.26 to 7.35 at 28°C; and 4.10 to 6.47 at 32°C, respectively (Table 2). The statistical analysis showed an increase in predation rates with temperature increase and prey density (Table 2). Six to seven prey at 24°C and ten prey at 28°C may thus be considered the plateau of the functional response, with the optimum temperature lying between 24 and 28 degrees.

**Table 2:** Mean predation rates (± standard errors) of *Mesostoma ehrenbergii* and *Mesostoma craci* on *Daphnia similis* at different temperatures and prey density combinations, over the period of 10 days.

Predator	Prey density	Temperature			
		20°C	24°C	28°C	32°C
***Mesostoma ehrenbergii***	**2**	1.92 (±0.05)a	1.82 (±0.05)a	2.00 (±0.05)a	1.95 (±0.05)a
	**5**	3.62 (±0.27)b	3.90 (±0.27)b	4.26 (±0.27)b	4.10 (±0.27)b
	**10**	4.02 (±0.55)b	5.05 (±0.55)b	7.35 (±0.55)c	6.47 (±0.55)c
***Mesostoma craci***	**2**	0.69 (±0.12)a	0.43 (±0.13)a	1.27 (±0.11)ab	Na
	**5**	1.15 (±0.17)ab	1.35 (±0.17)abc	2.55 (±0.17)cd	Na
	**10**	0.92 (±0.25)ab	1.76 (±0.25)bc	3.25 (±0.25)d	Na

Different letters mean consumption differences at different temperatures and predation rates (p <0.05).

Na: not available.

The mean consumption of *M*. *craci* at the lowest prey density was 0.69 at 20°C; 0.43 at 24°C and 1.27 at 28°C. At higher prey densities (5 and 10), consumption corresponded to 1.15 and 0.92 at 20°C; 1.35 and 1.76 at 24°C; and 2.55 and 3.25 at 28°C, respectively (Table 2). There was an increase in predation rates at the highest temperature and prey density at higher temperatures. *M*. *craci* did not survive at 32°C and performed poorly at 20°C with consumption independent of the amount of food offered. It may thus be classified as a stenotherm.

**Fig 2:**
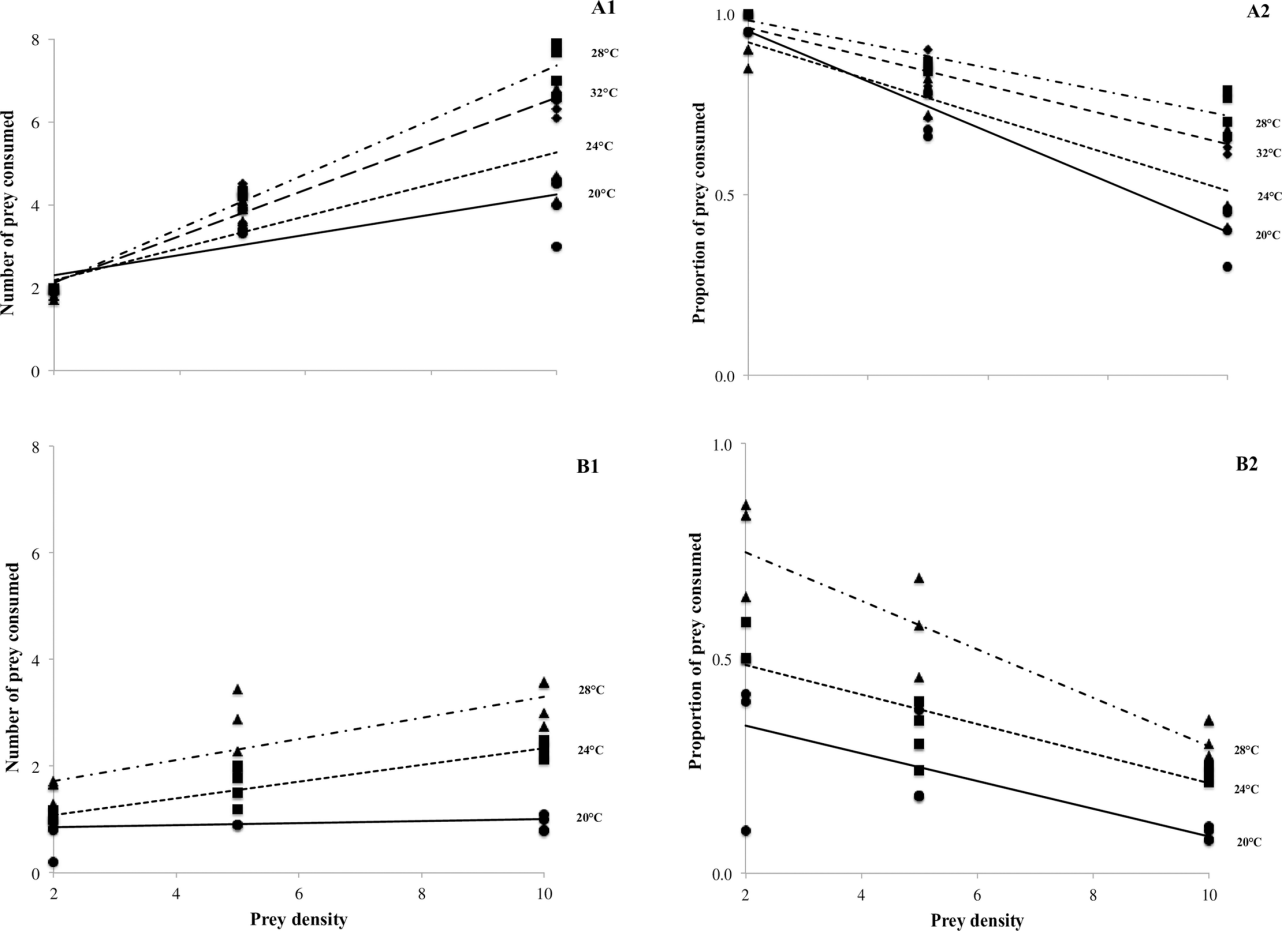
Prey consumed as function of prey density (A_1_ and B_1_) and prey consumed/prey offered ratio (A_2_ and B_2_) for *Mesostoma ehrenbergii* and *Mesostoma craci*, respectively. Closed circle, closed square, closed triangle and closed diamond correspond to 20, 24, 28 and 32°C, respectively.

*M*. *ehrenbergii* showed higher consumption as prey density increased, showing a type II functional response. Predation also increased with temperature, except at 32°C, when it was lower than at 28°C (Fig 2A_1_). At 20°C, daily predation by *M*. *craci* was similar for all prey densities, varying from 1 to 2 prey consumed per day (Fig 2B_1_). At 28°C, daily feeding increased with prey density, with higher predation rates at higher densities, also showing a type II functional response. The increase in prey consumption was more evident at 28°C. At 20 and 24°C there was slight variation (Fig 2B_1_).

An inverse relation between prey consumption ratio and prey density was found in both predators (Fig 2A_2_ and 2B_2_). In *M*. *ehrenbergii*, the slope was lower at 32°C than at 28°C.

**Fig 3:**
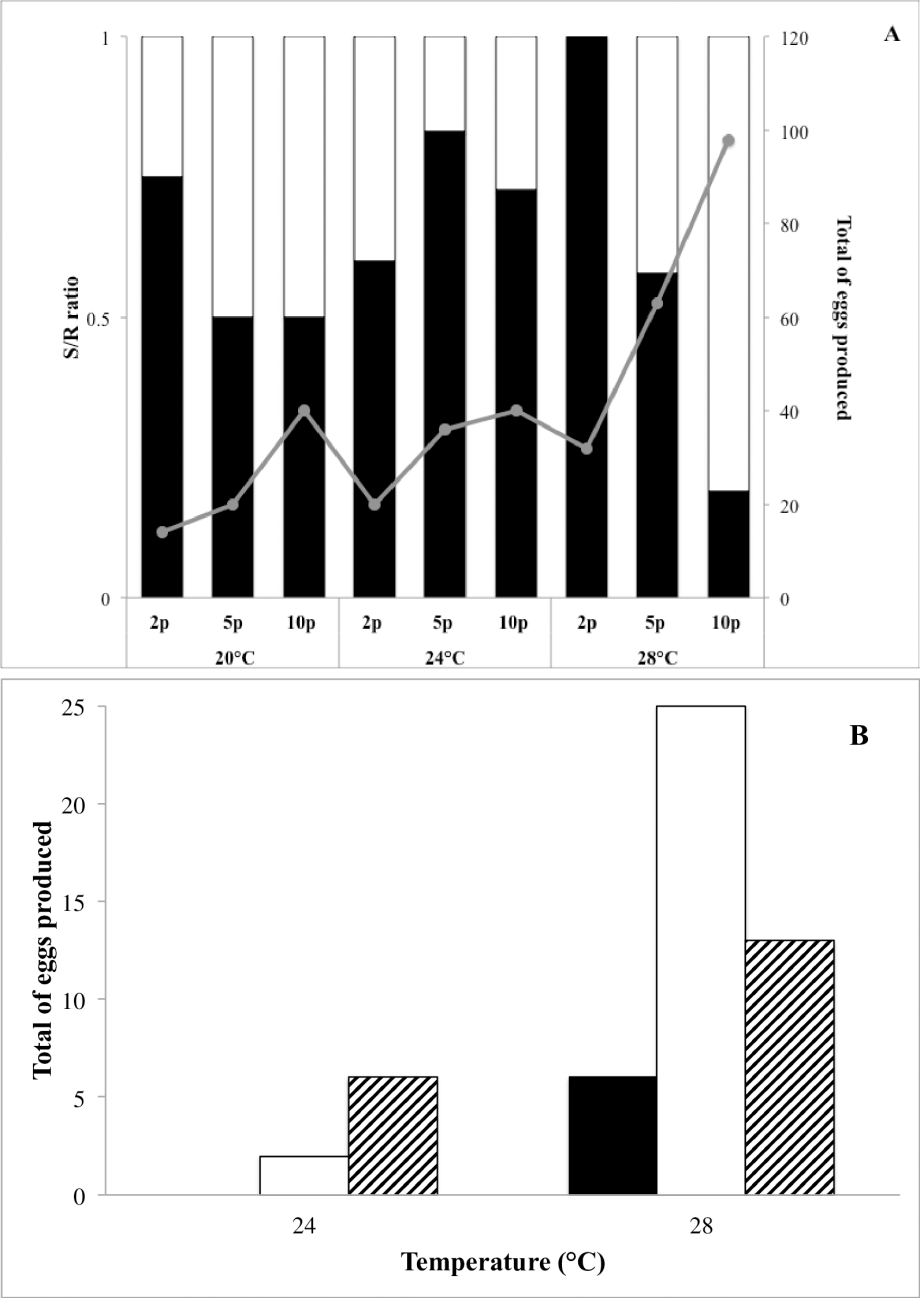
Proportion of subitaneous and resting eggs in *Mesostoma ehrenbergii* (A) where open bars, filled bars and filled circles correspond to subitaneous eggs, resting eggs and total number of eggs; and (B): total number of eggs produced by *Mesostoma craci* at different temperatures and prey densities. Filled, open and dashed bars correspond to 2, 5 and 10 prey.

Fig 3A presents the proportion of subitaneous and resting eggs of the first clutch of *M*. *ehrenbergii* at 20, 24 and 28±1°C and the total number of eggs produced across 10 days. Subitaneous and resting egg production was independent of temperature and food. At 32°C, only one turbellarian produced 2 resting eggs at the highest prey density. On the other hand, temperature (p<0.05, F = 4.57, df = 2) and prey density (p<0.01, F = 72.83, df = 2) daily offered showed a significant effect on the total egg production for *M*. *ehrenbergii*. More eggs were produced with 10 prey (40,40 and 98) at 20, 24 and 28°C, respectively, followed by 5 prey (20, 36 and 63) at 20, 24 and 28°C, respectively. When only 2 prey were offered, fewer eggs were produced (14, 20 and 32) at 20, 24 and 28°C.

*M*. *craci* did not produce true resting eggs at any temperature or food condition. A kind of pseudo-resting (or pseudo-subitaneous) eggs were released, that hatched within a week. Fig 3B shows its egg production at 24 and 28±1°C. No eggs were produced at 20°C and only at 28°C there was egg production at all prey densities. Total eggs produced increased with temperature (p = 0.01, F = 3.75, df = 1). However, no differences of egg production related to prey density (p = 0.18) or to an interaction of both variables were found (p = 0.24).

**Fig 4:**
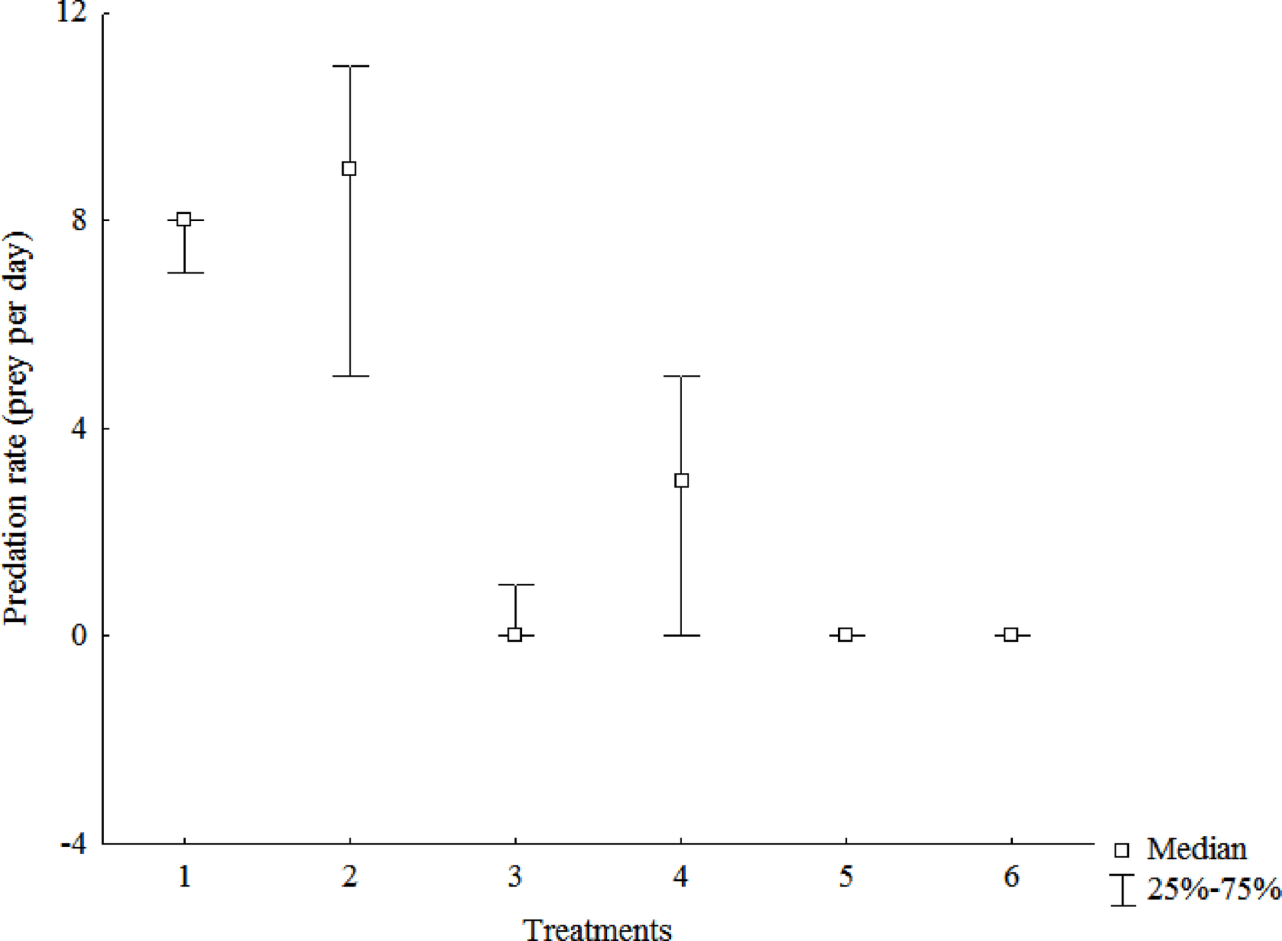
Predation rates of *Mesostoma ehrenbergii* on *Mesostoma craci*. The box represents the 25 and 75% quartiles and the band in the box is the median value.

In the intra-*Mesostoma* experiment, no predation of *M*. *craci* on *M*. *ehrenbergii* was observed in any treatment while *M*. *ehrenbergii* preyed on *M*. *craci* in treatments 1 to 4 (H = 14.32; p = 0.014) (Fig 4). In treatments 1 and 2, adult *M*. *ehrenbergii* median consumption corresponded to 8 and 9 prey. In treatment 3, one young *M*. *ehrenbergii* consumed one young *M*. *craci* in one of the three replicates. In treatment 4, young *M*. *ehrenbergii* median consumption corresponded to 3 *M*. *craci* individuals.

Eggs of *M*. *craci* in control and treatments did not differ in hatching percentage (87.5% and 80.9%, respectively, p = 0.08, X^2^ = 2.99, df = 1) and time (4.3 and 3.0, respectively, p = 0.12, t = 1.60, df = 1). However, eggs released in cultures at 24±1°C, collected and dried and rehydrated, did not hatch. In normal culturing conditions, hatching was over 70%.

## Discussion

Mesostomine flatworms mainly occur in the pelagic of water bodies that lack a proper littoral zone and have reduced limnetic but abundant littoral fish populations [24]. In such cases, they prey upon the crustacean plankton, often with a preference for daphnids [17, 30, 31]. If more than one species of flatworm inhabits the plankton, the larger one may prey on the smaller one, as experimentally shown in the present study.

Despite the relevance of the subject, it has not been investigated in co-occurring congenerics. Laboratory experiments have documented predation effects of *M*. *ehrenbergii* on cladocerans [35, 36]. Much less information is available on *M*. *craci*. Rocha et al. [21] provided field data on predation rates of *Mesostoma* sp, later identified as *M*. *craci* (Noreña, personal communication). They reported a preference for cladocerans (2–5 prey/flatworm per day), with rates similar to those reported for *M*. *lingua* in the lab under similar temperatures by Dumont and Schorreels [37].

Although *M*. *craci* is much smaller than *M*. *ehrenbergii*, young specimens (few days old) could consume neonates of *Daphnia similis*. Additional experiments at 24*±*1°C at prey densities similar to this study (S2 Table), considering the first three days of predation of young *M*. *craci* on neonates of *D*. *similis* and *Ceriodaphnia silvestrii* (a much smaller prey, around 1/3 of the first) did not show differences (p = 0.80). In later life, it was mainly temperature that influenced predation rates. In nature, small prey including protozoans and rotifers are probably consumed more than cladocerans. Núñez-Ortiz et al. [38] showed a preference for rotifers in the small *Stenostomum leucops* as well, with a functional response close to that here found for *M*. *craci*. On the other hand, *M*. *craci* may not prey daily. Although 2 to 5 prey were generally enough for the species, its feeding rates may have been masked by this feeding gap, thus not providing a linear mixed fit as good as the obtained for *M*. *ehrenbergii*.

In *M*. *ehrenbergii*, all stages of daphnids occurred in its diet. At the lowest density, all available prey were consumed. At other prey densities, consumption peaked after 6–7 days age, when energy allocation for egg production was required. The experiments confirmed that *Mesostoma* consume more food as temperature rises, and that they eat more as prey become more common. This leads to a functional response of type II, especially clear in *M*. *ehrenbergii*. In *M*. *craci*, at 20°C the predation numerical response collapsed, while at 32°C the species did not survive, making it the more stenotherm of the two.

Eggs of both turbellarians began to be produced after about a week. However, the total number of eggs produced by *M*. *ehrenbergii* showed differences related to temperature and prey density combinations, with more eggs being produced at higher temperatures and prey densities. *M*. *craci* only reproduced at 24 and 28°C, showing differences in total fecundity related only to temperature.

As to their somatic growth from 20 to 28°C (S2 Fig), 28°C was the more favorable temperature (p = 0.00) for *M*. *ehrenbergii*, especially at higher prey densities (p = 0.00). The lower temperatures (20 and 24°C), did not result in differences in somatic growth. In the case of *M*. *craci*, somatic growth did not differ at the three temperatures tested across 10 days (p = 0.35). Also, no differences were found related to prey density (p = 0.43). Being a smaller- sized species of *Mesostoma*, smaller numbers of *D*. *similis* were required to satiate it.

We recorded whether temperature and prey influenced the type of eggs produced. At first reproduction, *M*. *ehrenbergii* showed no pattern. A mix of subitaneous and resting eggs was produced in the first clutch, as in the studies by Heitkamp [26]. The subtropical population of Brazil thus behaves in the same way as populations from temperate Germany. From the second clutch on, under whatever conditions, only resting eggs were produced, again like in the German populations. Thus, *M*. *ehrenbergii* shows this strategy for reproduction independent of temperature and food.

It remains possible that the mix itself is influenced by food and temperature: such an effect was unclear at 20° and 24°C, but at 28°C, the more food was offered, the fewer resting eggs were produced. Experiments on the life cycle at *ad libitum* conditions (S3A Fig), showed that resting eggs accounted for 12% and 26% of total egg production at 20 and 24°C but 44% at 28°C and 50% at 32°C, respectively. Thus, 20–24°C did not have much impact on the reproductive strategies of *M*. *ehrenbergii* but temperatures higher than that did.

Heitkamp documented [39] climatic effects in the north of Germany. One extreme is the (sub) polar *M*. *nigrirostrum* that produces only one clutch of all-resting eggs per year. In our case, the highest temperature tested also showed such effects. Moreover when same prey densities were kept during their life cycle at 32°C (around three weeks, S3B Fig), only 6 subitaneous non-viable eggs at the lower prey density and 2 resting eggs at the highest prey density were produced by *M*. *ehrenbergii*. At *ad libitum* feeding, only 3 out of 10 individuals reproduced. Total fecundity was low (52 eggs), represented by 21 subitaneous eggs (mostly non-viable) and 31 resting eggs.

Additionally, experiments considering different volumes and densities of *M*. *ehrenbergii* reported by Dumont et al. [24], showed not only effects on resting egg production but also revealed that subitaneous eggs, becoming rarer as population density increased, did not necessarily hatch. Thus, beside climatic and density-dependent effects on resting egg production [40,41] another trigger in the reproductive strategy of *M*. *ehrenbergii*, was self- inhibition (subitaneous but non-viable eggs), at high abundances of its own population.

The situation in *M*. *craci* was different: here, an intermediate type of egg was produced. These pseudo-resting or pseudo-subitaneous hatched spontaneously after about one week, against several months for resting eggs of *M*. *ehrenbergii*. On the other hand, in experiments with *M ehrenbergii* preying on *M*. *craci*, viable eggs of *M*. *craci* eaten by *M*. *ehrenbergii* passed the digestive tract unharmed, and hatched few days later. This property is shared with true resting-eggs. However, the eggs failed to hatch after desiccation. They seem to share this property with subitaneous eggs that go through quiescence as described by Katajisto [42].These findings have not yet been recorded in turbellarians.

As to the influence of *Mesostoma* on zooplankton community structure, the predation rates here verified may suppress cladoceran species, beside a variety of freshwater invertebrates such as mosquito larvae, protozoans, rotifers, and copepods [17, 23]. This effect becomes stronger as the climate becomes warmer, and may explain why pelagic typhloplanids become more common towards the tropics.

*M*. *ehrenbergii* consumed around 2 prey per day regulating zooplankton population dynamics in western Colorado ponds, as reported by Maly et al. [17]. This rate is similar to that experimentally found by Dumont and Schorreels [35] in the related *M*. *lingua*. Here, *M*. *craci* reached predation rates up to 1.4 daphnids per day, at optimum temperature and prey density. This is somewhat lower than the maximum rates reported by Schwartz and Hebert [18] for *M*. *lingua* (2.0 daphnid per day). In the case of *M*. *ehrenbergii*, predation rates were even higher (7 prey per day at 24–25°C), which should affect on the population dynamics of cladocerans (mainly represented by daphnids) in their natural environment [24,28].

With respect to feeding habits, cannibalism often occurs when food is in short supply [43]. Although this was reported more than half a century ago, it has not made its way into recent literature on flatworms ecology. In our study, the large *M*. *ehrenbergi*, preyed upon adult but small *M*. *craci*, at high rates, independent of prey density. A lower number of prey was consumed when young *M*. *craci* were offered, underscoring *M*. *ehrenbergii*’s preference for large-sized prey. By this relaxed predation on young *M*. *craci*, it might survive in co-existence. Reynoldson [44, 45] and Reynoldson and Davies [46] studied the co-existence of the congeneric *Polycelis nigra* and *Polycelis tenuis*, finding overlap in diet. Reynoldson and Davies [46] concluded that co-existence was achieved by food partitioning, each one eating more of a different type of prey, later termed food refuge [47]. Also, the reproductive strategy of both species and especially of *M*. *ehrenbergii*, which produces a higher number of eggs, seems to minimize intra-specific predation and risk of extinction [48].

## Conclusions

*M*. *ehrenbergii* and *M*. *craci* predation rates fitted a linear mixed-effects model and showed a type II functional response. Growth increased with temperature, more prey were consumed and more eggs produced. *M*. *craci* was stenothermic, *M ehrenbergii* eurythermic.

*M*. *ehrenbergii*, a large species and a cladoceran feeder coexisting with *M*. *craci* (a smaller species) in their lake of origin, fed on the smaller flatworm but not vice versa, even when adult *M*. *craci* were confronted with young *M*. *ehrenbergii*.

*M*. *ehrenbergii* produced subitaneous eggs at first reproduction only, independent of food and temperature, and resting eggs afterwards. This may represent a mechanism for controlling population size.

*M*. *craci* produced an intermediate type of egg, combining characteristics of subitaneous and resting eggs. These eggs hatch shortly after being released, remain viable after passage through the gut of *M*. *ehrenbergii*, but do not resist drying as do true resting eggs.

## Supporting information

S1 Fig Daily consumption of *Daphnia similis* by *Mesostoma ehrenbergii* (A1-12) and *Mesostoma craci* (B1-9) under different temperatures and prey densities during 10 days. Curves were fit by linear mixed-effects models.
(TIF)

S2 Fig Somatic growth of *Mesostoma ehrenbergii* (A) and *Mesostoma craci* (B) at different temperatures (20, 24 and 28°C) and prey densities during 10 days, including initial, mid and final measurements (filled circles, squares and triangles correspond to 2, 5 and 10 prey, respectively).
(TIFF)

S3 Fig Proportion of resting eggs at different temperatures and *ad libitum* feeding (A) and total number of eggs produced at different prey densities (B) in *Mesostoma ehrenbergii*. Open and filled bars correspond to subitaneous and resting eggs, respectively.
(TIF)

S1 Table Statistical results (p-values, F and R2) of the linear mixed-effects models for *Mesostoma ehrenbergii* and *Mesostoma craci*. Na: not available.
(TIF)

S2 Table Predation rates of *Mesostoma craci* on *Daphnia similis* (present study) and on *Ceriodaphnia silvestrii* (additional experiment) during 3 days, and a comparative statistical analysis. (TIFF)
