# Supplementary figures and images for: Predation and reproductive performance in two pelagic typhloplanid turbellarians
Source: PLoS One. 2018 Mar 14;13(3):e0193472. doi: 10.1371/journal.pone.0193472 (PMC5851573; doi:10.1371/journal.pone.0193472)

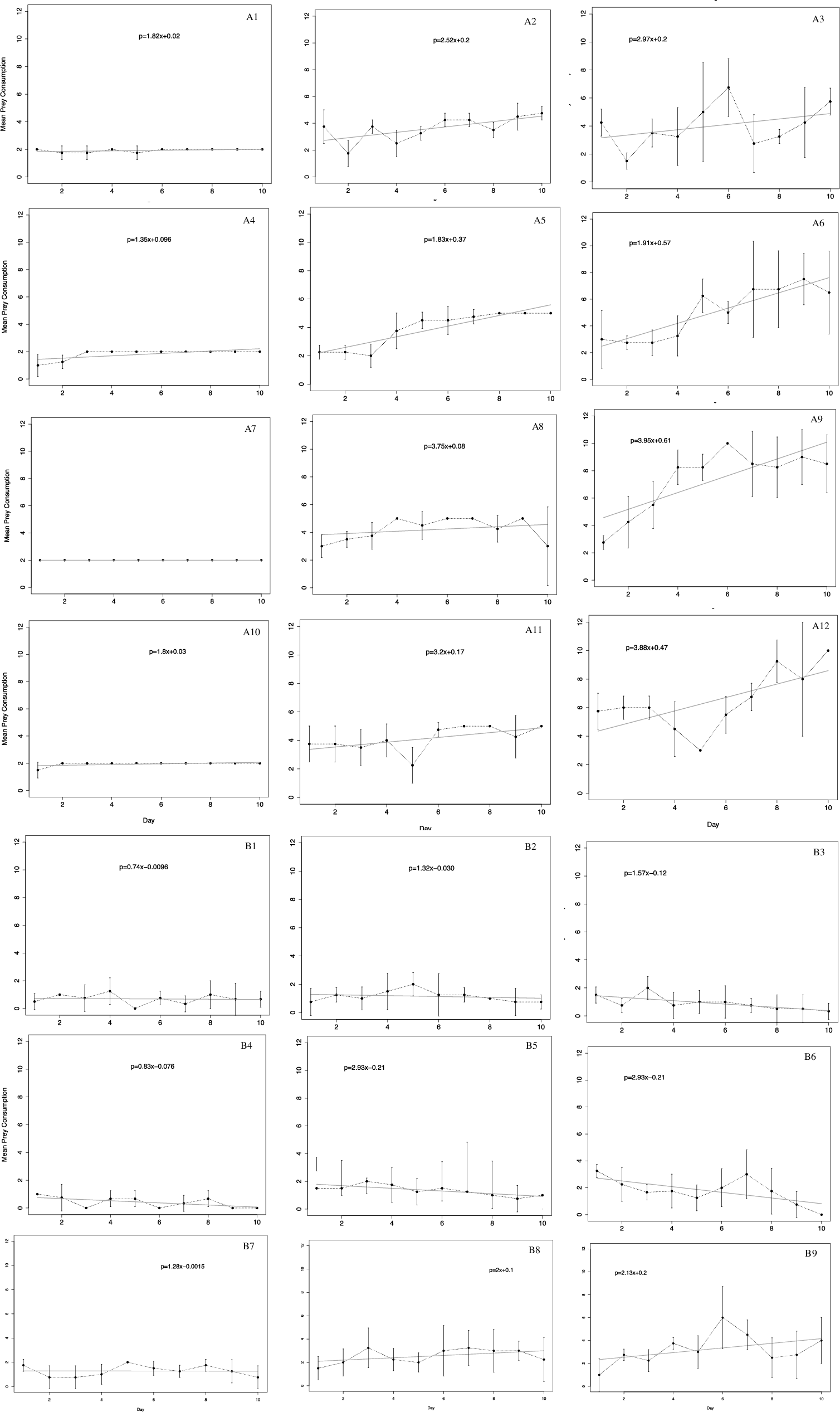

Supplement: S1 Fig — Daily consumption of Daphnia similis by Mesostoma ehrenbergii (A1-12) and Mesostoma craci (B1-9) under different temperatures and prey densities during 10 days. Curves were fit by linear mixed-effects models. (TIF) [file pone.0193472.s001.tif]

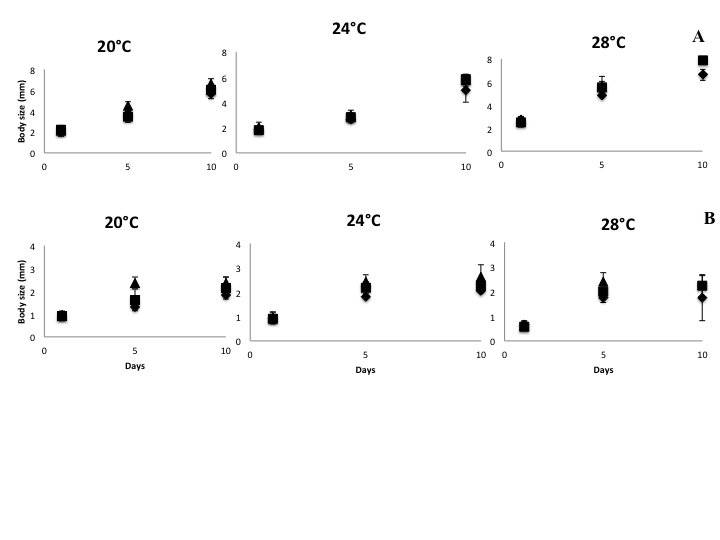

Supplement: S2 Fig — Somatic growth of Mesostoma ehrenbergii (A) and Mesostoma craci (B) at different temperatures (20, 24 and 28°C) and prey densities during 10 days, including initial, mid and final measurements (filled circles, squares and triangles correspond to 2, 5 and 10 prey, respectively). (TIFF) [file pone.0193472.s002.tiff]

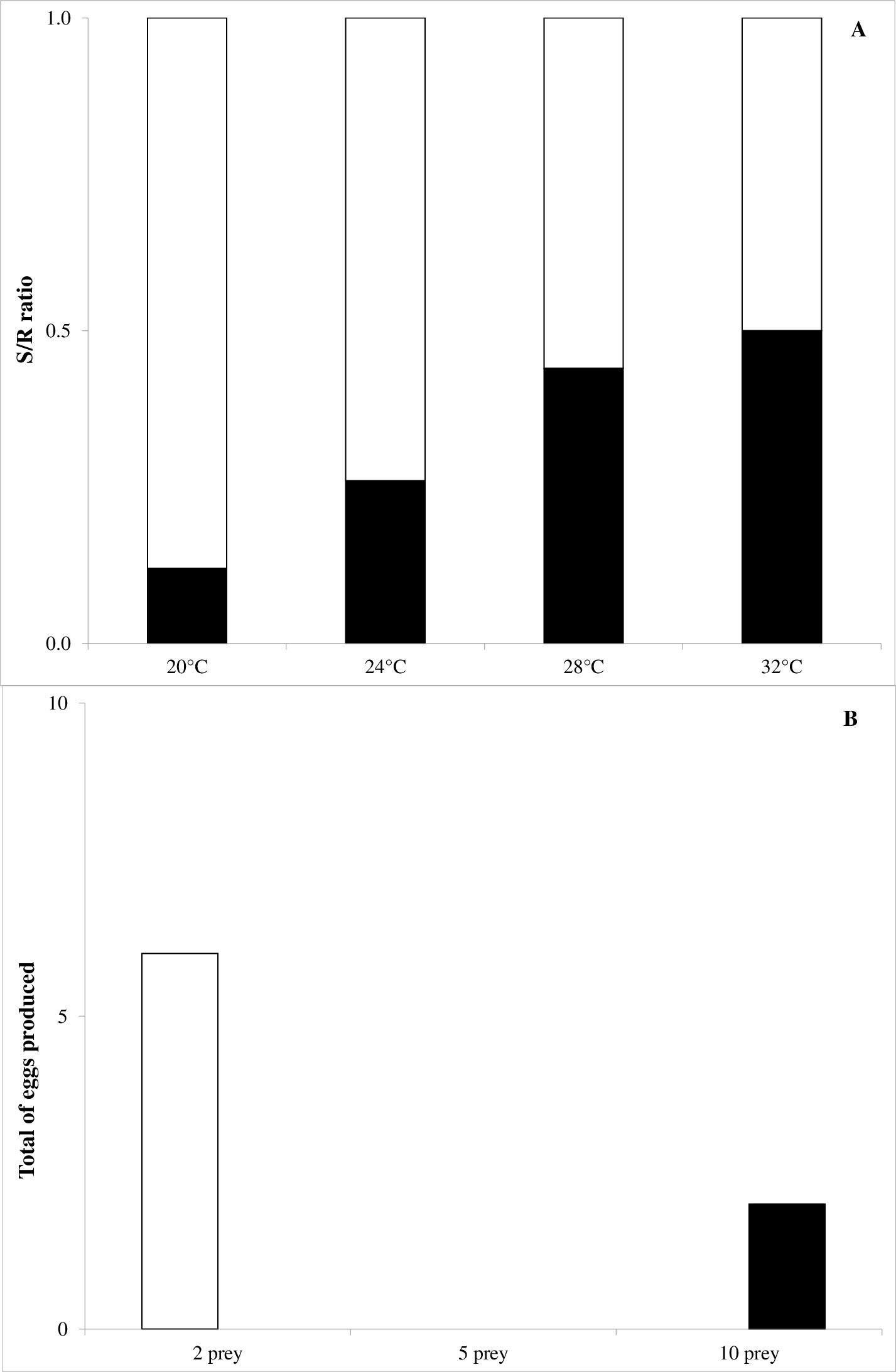

Supplement: S3 Fig — Proportion of resting eggs at different temperatures and ad libitum feeding (A) and total number of eggs produced at different prey densities (B) in Mesostoma ehrenbergii. Open and filled bars correspond to subitaneous and resting eggs, respectively. (TIF) [file pone.0193472.s003.tif]

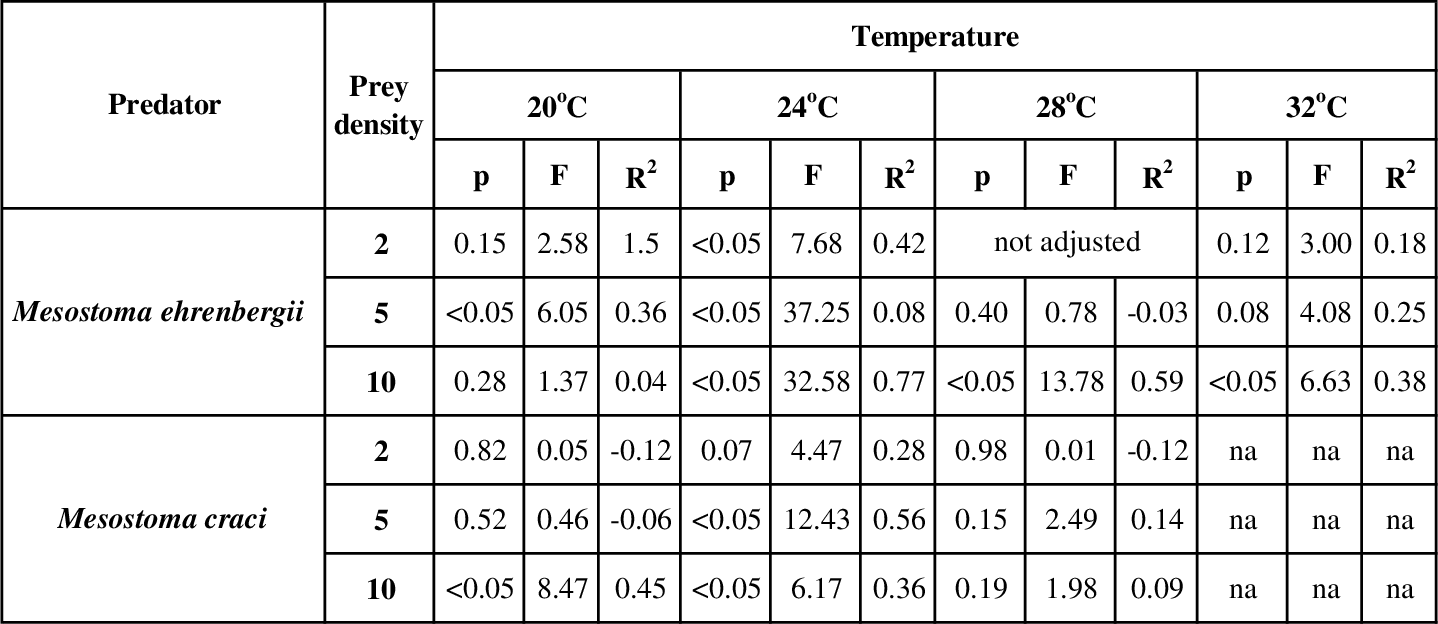

Supplement: S1 Table — Na: not available. (TIF) [file pone.0193472.s004.tif]

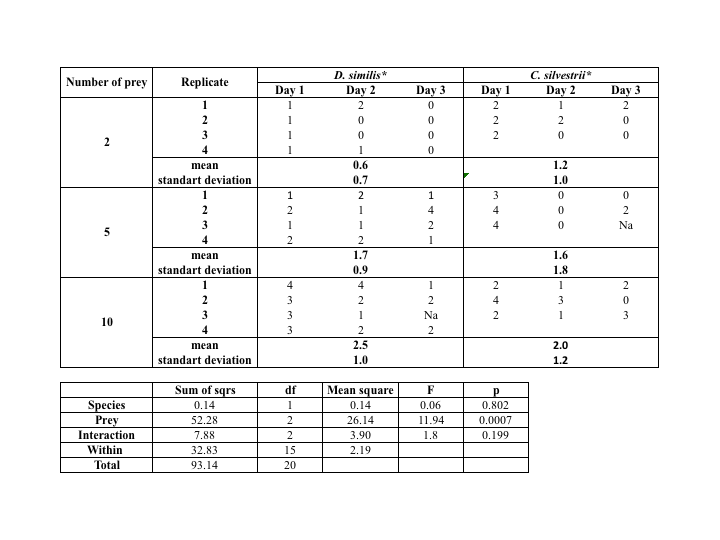

Supplement: S2 Table — (TIFF) [file pone.0193472.s005.tiff]
